# The Effect of Osteopathic Manipulative Treatment Adjunct on Stabilization Splint Treatment in Temporomandibular Joint Anterior Disc Displacement with Reduction Disorder: A Quantitative Analysis, Pilot Study

**DOI:** 10.3390/jcm14082544

**Published:** 2025-04-08

**Authors:** Ayça Aklar, Burcu Bal, Neslihan Taşdelen, H. Serap İnal, Gökhan Ertaş

**Affiliations:** 1 Physiotherapy and Rehabilitation Department, Başkent University Altunizade Hospital, Istanbul 34662, Turkey; 2 Department of Prosthodontics, Faculty of Dentistry, Yeditepe University, Istanbul 34730, Turkey; 3 Department of Radiology, Faculty of Medicine, Yeditepe University, Istanbul 34638, Turkey; 4 Department of Physiotherapy and Rehabilitation, Istanbul Galata University, Istanbul 6290, Turkey; 5 Department of Biomedical Engineering, Faculty of Engineering, Yeditepe University, Istanbul 34640, Turkey

**Keywords:** temporomandibular joint, temporomandibular joint disk, osteopathic manipulative treatment, magnetic resonance imaging

## Abstract

**Objective:** This study aimed to quantitatively assess the efficacy of osteopathic manipulative treatment (OMT) as an adjunct to stabilization splint treatment for temporomandibular joint anterior disc displacement with reduction (ADDwR) disorder. **Method:** Thirty-eight joints were evaluated in this study. OMT, in addition to splint treatment, was administered to the study group, and the control group only received splint treatment. Before and after the treatments, the quality of life, the pain levels of distinct body regions, and the sleep quality were evaluated, while mandibular movements were assessed and condyle–disc position differences measured on magnetic resonance images. **Results:** In all the life qualities, except social functionality, increases after treatment were present for both control and study groups, but the increase was significant in bodily pain for the control group (*p* = 0.034) while increases were significant in physical functionality (*p* = 0.018), general health perception (*p* = 0.027), and reported health transition (*p* = 0.019) for the study group. Significant pain reduction in all body regions was seen in the study groups (*p* = 0.007–0.033), but the decrease was only significant for the temporomandibular joint for the control group (*p* = 0.011). The sleep quality significantly improved for the study group (*p* = 0.018). Limited after-treatment improvements in the condyle–disc positions were determined for both the normal joints and joints with ADDwR in the study group. **Conclusions:** The application of OMT adjunct to splint treatment increases quality of life and sleep, reduces bodily pain, and improves condyle–disc positions over the MRIs of TMJ.

## 1. Introduction

Temporomandibular disorder (TMD) is a wide-ranging terminology that involves dysfunction within the muscles of mastication, the intra- or extra-articular structures of the temporomandibular joint, and related structures. The most common symptom of this disorder is pain located over the masticatory muscles or pre-auricular region; other symptoms include restricted mandibular movements, joint sounds (click or crepitus), and difficulty in speaking [1]. The disorder includes musculoskeletal problems that may cause pain in the orofacial region. Recently, several studies were conducted to explain the etiology, signs, epidemiology, and progress of the disease and categorized the disorder under “psychosocial musculoskeletal disorders with a multifactorial etiology” [2,3,4,5,6,7,8,9]. Biomechanical, neuromuscular, neurobiological, and psychosocial factors may lead to the development of this disorder. In addition to the multifactorial etiology, trauma, parafunctions, joint hypermobility, and stress, certain types of personality may potentially contribute to the progression of the disorder. The incidence of TMD varies widely, ranging 34% [3]. Therefore, TMD is considered to be the second most common musculoskeletal disorder causing pain and physical disability [4]. In addition, disc displacement and degenerative disorders of the temporomandibular joint are commonly observed in approximately 19.1% and 9.8% of cases, respectively [5]. Disc displacement is classified as an intracapsular disorder involving the disc–condyle complex. If the disc is in an anterior position compared to the mandibular condyle in a closed-mouth position and is aligned with the condyle after opening the mouth, it is called anterior disc displacement with reduction, usually accompanied by clicking, popping, and snapping noises from the joint [6]. This disorder is clinically diagnosed with supporting findings from magnetic resonance (MR) images of the closed- and open-mouth tasks of the joint.

As TMD is a term that is used for a combination of multifactorial dysfunctions, for its treatment, distinct methods can be proposed by dentists, orthodontists, psychologists, physical therapists, osteopaths, and physicians, but interdisciplinary methods are increasingly used [7]. Smith et al. conducted a study in 2013 showing the genetic variants involved in the development of TMD [9]. Among a large sample size, this study revealed that there is no significant incidence of genetic changes in developing TMD. A recent study investigating the etiological factors of TMD showed that TMD is commonly established with biopsychosocial factors [10]. Comorbid conditions such as irritable bowel syndrome and insomnia are strong predictors of TMD onset. In addition, some evidence shows the relation between malocclusion and parafunctional habits [11].

These methods can be categorized as noninvasive and invasive. Noninvasive methods usually use muscle relaxants or anti-inflammatory medicines, oral splints, home exercises, cognitive-behavioral information programs, acupuncture or dry needling, physical therapy, osteopathic manipulative treatment, relaxation exercises, or meditation. All these efforts aim to relieve musculoskeletal complaints, reduce inflammation, and restore the motor function of the mastication system. Invasive methods, such as arthrocentesis, Botox treatment, injections, arthroscopy, and condylotomy, are used when noninvasive treatments become ineffective [12].

Osteopathic manipulative treatment (OMT) is a manual medicine invented by Andrew Taylor Still in 1902. Still hypothesized that OMT relies on the body’s ability to heal itself, appreciating the unity of the body, mind, spirit, and the myofascial connections between structures and myofascial force transmission within structures [13]. Differing from physiotherapy and massage, osteopathy emphasizes the whole body. By evaluating the body units, osteopaths differentiate and treat the main structure causing the symptoms of the patient, which could be further away from the complained zone. A spinal cord segment makes an afferent and efferent nerve connection at the dorsal, anterior, and lateral horns. Those connections may be related to the sensory-motor information of the visceral organs, muscles, fascia, dermatomes, arteries, skeleton, and other neurological structures. Therefore, from the same spinal cord level, multiple structures are governed. In OMT, all these structures related to the spinal cord segment, according to the tissue referral, should be evaluated and treated when necessary [14,15].

Emerging evidence suggests that OMT may have a significant effect on headaches, migraines, and cranial pain [15]. Although expectations for pain-relieving treatment are notable, and the treatment has been shown to be effective in reducing pain and increasing the mandibular range of motion, there is a lack of quantitative analyses, especially for the disc–condyle positions for TMDs [15,16,17,18,19]. Moreover, the mechanical outcomes of disc displacement treatment with the reduction subtype of the disorder remain unclear. The current study aimed to quantitatively investigate the efficacy of osteopathic manipulative treatment for temporomandibular joint anterior disc displacement with reduction disorder by performing a randomized controlled trial in which the efficiency of a stabilization splint treatment combined with an osteopathic manipulative treatment (SS + OMT) was compared with the efficiency of a stabilization splint treatment alone (SST), considering the following hypotheses:

**H0.**  *SS + OMT does not cause any improvement in quality of life, pain, sleep quality, or cervical and mandibular movements when compared to SST.*

**H1.**  *SS + OMT causes an increase in quality of life, a decrease in pain, an increase in sleep quality, and improvements in cervical and mandibular movements when compared to SST.*

## 2. Materials and Methods

### 2.1. Study Population

This study was conducted with the approval of the Yeditepe University Clinical Research Ethics Committee (16.05.2018/No:846) and the Ministry of Health of Turkey (18.07.2018/No: E.201129), according to the declaration of Helsinki. G-power software, which is used to calculate the power analysis, determined the sample size as 30 participants. Due to the pandemics, patient gathering and data acquisition had to stop in March 2020. A total of thirty-six patients were considered with temporomandibular joint pain and clicking sound, diagnosed with disc displacement without degenerative arthritis at Yeditepe University Dental Hospital. They were not receiving orthodontic treatment or antidepressant medications, had no history of systemic diseases, no pain due to oral or dental pathologies, no dental implant placement, no exodontia within six months, no cranial and cervical pathology, no trauma history or surgery; and were not showing contraindication to MR imaging. Diagnostic criteria for TMJ disorders and MRI were used to diagnose and differentiate the underlying pathology. The patients were informed about the lifestyle habits protecting the temporomandibular joint structures, the traditional treatment of the disorder using stabilization splints, and the details of the osteopathic manipulative treatment planned for the disorder. Thirty-six patients were evaluated at the Yeditepe University Dental Hospital, of which 10 patients were excluded from the study due to subluxation and degenerative changes of the TMJ, and one patient was excluded for undergoing masseter Botox treatment. A total of 24 patients were diagnosed with ADDwR, of which 21 patients (42 joints) volunteered to participate in the study and provided written informed consent. These patients were evaluated at the Yeditepe University Radiology Department. Occlusal Splints were manufactured for all patients. All patients were informed about TMD diseases and lifestyle changes. Thereafter, 21 patients (28 joints) were randomly assigned to a “control group” and a “study group” using the sealed envelope method. As the sample size was small, an equal number of written papers were put in envelopes, and patients were asked to choose one to determine the groups. Except for the osteopath who gave the patients treatments, all the evaluators were blind to the groups. Two patients were excluded because of an issue with occlusal splint usage. The control group (n = 9) received SST, while the study group (n = 10) received SS + OMT.

### 2.2. Treatment Plans

Patients in the control group received SST that involved the use of an occlusal stabilization splint during sleep for six weeks. Tailored mandibular splints were manufactured and customized to have maximal posterior bilateral contact in centric occlusion and complete anterior contacts during protrusive movement of the mandible and canine guidance during lateral mandibular movement.

**Figure 1:**
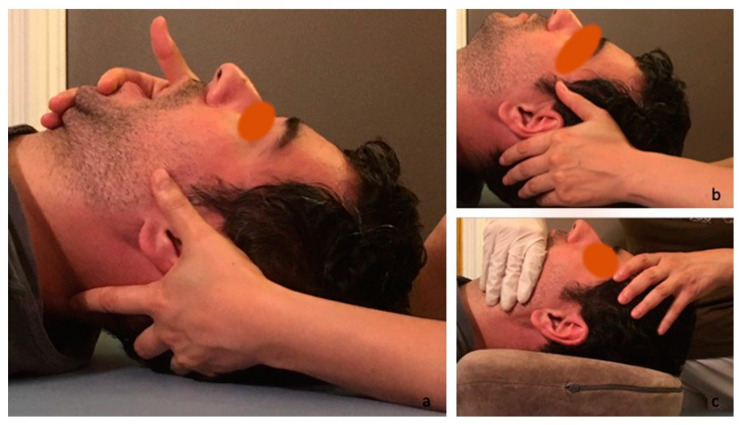
Treatment of (**a**,**b**) muscular patterns and (**c**) articular structures.

The patients in the study group received SS + OMT, wherein osteopathic manipulative treatment was applied twice a week by a physiotherapist specializing in osteopathy in addition to splint treatment for six weeks. The physiotherapist evaluated the cranium, TMJ motions, pain levels, specific muscles, and joints to assess the origins and levels of dysfunction advocated by the jaw joints and the postural patterns before performing osteopathic manipulations that involved the application of gentle pressure and releasing techniques over fascial restrictions and joint mobilizations in a fixed order lasting 30–40 min in accordance with the procedures described in the literature [14]. During osteopathic manipulative treatments, Grade I or II mobilization was applied to restricted movements of the mandible. For the TMJ mobilizations, the force was applied in the direction to distract the condyle and increase the condyle’s arthrokinematic motions such as gliding and rotatory movements (Figure 1). To relax the muscles and desensitize the pain around the jaw, intra- and extra-oral muscle relaxation techniques were applied to the masseter, lateral pterygoid, medial pterygoid, infrahyoid, and temporal muscles to reduce muscular tension, whereas post-isometric relaxation was applied around the masticatory muscles during mouth opening, mouth closing, protrusion, retrusion, and lateral excursions. In addition, fascial mobilization techniques and Grade I or II mobilization were applied to further restrict the musculoskeletal areas of the body, which were evaluated and noted at the beginning of treatment. The treatment was emphasized in the craniocervical, thoracic, and sacral regions because of the known fascial connections of the temporomandibular joint [20]. In addition, considering the sensation and nociceptive innervation of the structures around the joint arising from the trigeminus, the neural pathways and the surrounding fascial structures were treated with special osteopathic manipulative techniques. Treatments were applied to the neural connections of the phrenic nerve (C3–5) and the trigeminus (where it joins to the spinothalamic tractus at the levels of C1–3), diaphragm, and upper abdominal organs. Rib raising and lymphatic pump techniques [21] were performed to balance the vegetative system and circulation.

Considering a joint (e.g., the temporomandibular joint) as a part of the body, with all the body parts (from very small to large) being mechanically and neurologically connected with each other, is always the focus of attention of osteopathy. Contrary to the accepted treatments of traditional medicine, osteopathy emphasizes both the joint itself and the neighborhoods of the joint while assessing and treating. When we consider the TMJ, there are functional links with the intracranial structures, such as falxcerebri and cerebelli and the tentorium cerebelli, and the continuing of these membranes within the cervical region nuchal muscles. These intracranial membranes spread the forces between the viscerocranium and the nuchal muscles. To balance the cranium and cervical region, the nuchal muscles compensate by changing their tonus, which may alter the occlusion patterns of TMJ [14].

A four-quadrant evaluation determined dysfunctions within the abdominal viscera. When tissue resistance occurred due to palpation, that area was considered dysfunctional. Visceral mobilization techniques were used fundamentally to the viscera, which has a mechanical or neurological relation with the fascial link arising from the temporomandibular joint (Table 1).

Evaluation of the temporomandibular joint was conducted while the patients were sitting. With one hand, the therapist fixed the patient’s head and held the lower jaw from the lower teeth and mandibular ramus. The therapist asked the patient to actively open, protrude, retract, and laterally excurse the lower jaw and at the end of the movement, to passively try to go further within that movement and for a second time, to passively check the whole movement. Mobilization was applied for the restricted parts in the spine lying position (Figure 1). For the repositioning of the anteriorly displaced disc, Farrar’s technique was used [14].

Before beginning and after completion of the treatments, which took 6 weeks, the patients filled in three questionnaires: the visual analog scale (VAS), the 36-item short-form health survey (SF-36), and the Pittsburgh sleep quality index (PSQI). VAS was used to assess the severity of pain in the temporomandibular joint, neck, chest, back, pelvis, and head. Pain was scored between 0 and 10 points, with a higher score indicating more severe pain. The SF-36 was used to reveal quality of life measures, such as physical functionality, social functionality, role limitation due to physical problems, role limitation due to emotional problems, mental health, energy and vitality, bodily pain, and general health perception. For each measure, a score was given between 0 and 100, with a higher score deemed a more favorable health quality [22,23]. The PSQI was used to determine sleep quality, giving a total score ranging from 0 to 21; higher values indicated worse quality [24].

### 2.3. Clinical and Radiological Findings

The temporomandibular joints and related body parts of the patients were examined clinically by a dentist and radiologically by a radiologist before and after treatment. The clinical findings of the patients were assessed at Yeditepe University Dental Hospital by a dentist with ten years of experience in TMDs. Routine examination methods were implemented to quantify the functional movements of the jaw, including mandibular elevation, depression, lateral excursion, protrusion, and retrusion, using a Vernier caliper. Lateral movement was measured from the midline of the upper and lower incisors while the patient was moving the mandible laterally from the midline to one side. The distance between the upper and lower incisors was measured during protrusion and retrusion without considering overbite or overjet [25]. The dentist assessed tender points over the masseter, lateral pterygoid, suprahyoid, and temporalis muscles, and palpated the upper trapezius and supraspinatus muscles, and the lateral epicondyle to eradicate fibromyalgia tender points over the sternocleidomastoids. The thumb pad of the dentist’s dominant hand was used to apply pressure to each muscle individually during the tender-point examination. Palpation was applied to the peri- and intra-auricular areas to detect pain due to joint inflammation [25].

**Figure 2:**
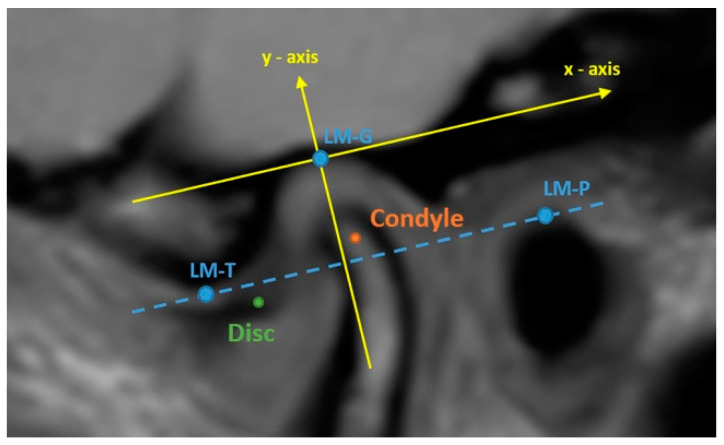
Drace–Enazmann’s methodology was applied to a proton density image. LM-T: the inferior border of articular tubercle. LM-P: the superior border of the porus acusticus externus. Dashed blue line: the line passing through LM-t and LM-P: LM-G: the highest point of the glenoid fossa.

The radiological findings of the temporomandibular joints of the patients were assessed using MR imaging by a radiologist who had specialized in musculoskeletal radiology for 20 years. MR imaging was performed at Yeditepe University Hospital using a 3T MR scanner with a 16-channel head coil and a dedicated imaging protocol. The protocol was initiated with a T1-weighted (TR/TE:450/8 ms) axial localizing scan to register the image slices acquired after treatment with those acquired before treatment. Next, T2-weighted (TR/TE:2600/80 ms) and proton-weighted (TR/TE:2000/21 ms) oblique sagittal scans vertical to the long axis of each condyle were performed. For all scans, the field of view was 150 mm ×150 mm, and the slice thickness was 3 mm. Images were gathered for the closed-mouth and maximally opened-mouth positions with the support of an MR-compatible device developed by our team. The radiologist browsed PD-weighted images to recognize the anatomy of the articular disc and adjunct soft tissue and T2-weighted images to detect synovial fluid and effusion. Meanwhile, the radiologist also inspected T1-weighted images for morphological and degenerative changes in the disc and condyle [26]. Subsequently, the radiologist performed quantitative angle and position measurements for the condyle and disc for the temporomandibular joint on three consecutive slices of the proton- and T2-weighted images in the sagittal plane for which the disc was clearly identified. For the measurements, Drace–Enazmann’s methodology was followed using OsiriX Lite tools: one landmark (LM-C) was placed at the center of the condyle head and another landmark was positioned at the midpoint of the posterior margin of the disc posterior band on the relevant images. A line connecting the landmarks and another line passing through the LM-C perpendicular to Frankfort’s horizontal plane were drawn. The disc–condyle angle was defined as the angle between the two lines. Disc–condyle angles from −15° to +15° were considered normal, while angles larger than +15° were treated for a disc with anterior displacement [27]. To measure the disc and condyle positions, two landmarks were positioned at the inferior border of the articular tubercle (LM-T) and the superior border of the porus acusticus externus (LM-P); an additional landmark was placed at the highest point of the glenoid fossa (LM-G) on the relevant images. A line connecting LM-T and LM-P was drawn. A line parallel to that line and passing through LM-G was drawn and assumed to be the reference “*X*-axis”. Another line was drawn perpendicular to this “*X*-axis” while passing through the LM-G and assumed to be the reference “*Y*-axis” (see Figure 2 for the illustrations). The condyle and disk positions were defined as the distances of LM-C and LM-D to these axes in millimeters. The angle and position measurement results from the three image slices for the closed mouth were averaged and used in the analysis.

### 2.4. Statistical Analysis

All statistical analyses were performed using IBM SPSS Statistics version 25 (IBM Corp., Armonk, NY, USA). Descriptive statistics were used to compare the quantitative parameters between the study and control groups. Significant differences in the continuous parameters were assessed using the Mann–Whitney U test or Wilcoxon signed-rank test. Fisher exact chi-square test and Fisher–Freeman–Halton test were used for categorical parameters. Cohen’s *d* was used to explain the power and effect size of the study, giving a total score ranging from 0 to 2, indicating the necessity of larger sample sizes and vice versa for lower values. A *p* < 0.05 was considered for statistical significance.

## 3. Results

Five of the 24 patients who entered the study dropped out due to misuse of the occlusal splint or reluctance to implement the treatment protocol. Consequently, the study was completed with 19 patients (age range—20–38 years; mean age—26.95 ± 6.87 years). The control group consisted of nine patients (2 men and 7 women), and the remaining ten were the study group (1 man and 9 women); the demographic data are listed in Table 2. Between the groups, no systematic difference was observed in the collected demographic attributes (*p* = 0.211–0.850) except for disease history (*p* = 0.020). The control and study groups included patients with similar age, height, weight, and social habits. However, eight patients had a history of digestive system and hormonal diseases. Their random assignment to the control and study groups resulted in a significantly higher number of patients with a history of disease in the study group compared to the control group. The patients’ clinical findings are presented in Table 3. There was no significant difference between the control and study groups in the clicking during mouth opening, occlusion, or bruxism (*p* = 0.076–0.430). Accordingly, the lowest pre-tendency was of concern in terms of both demographic attributes and joint findings for the study population.

Pre- and post-treatment quality of life data gathered by the SF-36 questionnaire and changes in the qualities determined are listed in Table 4. Insignificant differences in quality of life before and after treatment were observed in both the control and study groups. However, for the control group, after treatment, increases were observed in most of the quality measures except social functionality, while the quality increase was significant in bodily pain (*p* = 0.034). For the study group, treatment increases were observed in all measures, but significant increases were only present in physical functionality (*p* = 0.018), general health perception (*p* = 0.027), and reported health transition (*p* = 0.019). When the changes calculated by subtracting the post-treatment values from the pre-treatment values for the quality-of-life measures were evaluated, significant differences were observed in physical functionality (*p* = 0.048) and reported health transition (*p* = 0.044). For SS + OMT, significant increases in the physical functionality, general health perceptions, and reported health transitions of the patients were of concern after the treatment. The pain scores collected by the VAS questionnaire before and after treatment and the changes in the scores determined for the temporomandibular joint, neck, back, lower back, pelvis, and head for the control and study groups are tabulated in Table 4. There were no significant differences in pain between the control and study groups before treatment. Still, there were significant differences in the temporomandibular joint pain and headache levels between the groups after treatment (*p* = 0.048 and *p* = 0.016, respectively). In the control group, pain reduction was detected; however, significant pain reduction was only observed in the temporomandibular joint (*p* = 0.011). In the study group, while there was a decrease in pain in all regions, significant reductions were detected not only in the temporomandibular joint but also in the neck, back, lower back, and head regions (*p* = 0.007–0.033). When the changes obtained by subtracting the post-treatment pain scores from the pre-treatment scores were examined, a significant difference was found between the control and study groups only for pain in the thoracic region (*p* = 0.018). When osteopathic manipulative treatment is applied in addition to splint treatment, there would be a decrease in the severity of especially joint pain and headaches; in addition to these, osteopathic manipulative treatment might effectively reduce the severity of the pain in the thoracic region after the treatment. The sleep quality before and after treatment assessed by the PSQI questionnaire and the change in the quality calculated are presented in Table 5. Some improvements in sleep quality were observed in the control group, although the differences were not significant. Conversely, there was a significant improvement in sleep quality in the study group (*p* = 0.018). However, while there was a significant difference in sleep quality between the control and study groups before treatment (*p* = 0.047), there was no significant difference after treatment. No significant changes were found between the control and study groups. The application of SS + OMT improved sleep quality, especially in individuals with low sleep quality (Table 6).

The clinical examinations before and after treatment revealed jaw and neck movements represented by the maximum mouth opening, mandibular protrusion, retrusion, and lateral excursions, cervical rotations, and cervical lateral flexions, listed in Table 7. For the control group, there were very limited increases or decreases in the range and angle of movements with no significance. However, in the study group, there were increases in the movement ranges and angles, and the increases in maximum mouth opening, mandibular retrusion and lateral excursions, cervical rotation, and lateral flexion were significant (*p* = 0.027–0.008). While there was a significant difference between the control and study groups only for left mandibular excursions before treatment (*p* = 0.030), a significant difference was observed between the groups after treatment only for cervical rotation on the left (*p* = 0.024). There were significant differences between the control and study groups in the changes in retrusion of the lower jaw, left neck rotation, and right–left neck lateral flexion (*p* = 0.021–0.002). Applying SS + OMT resulted in an improved range of motion for the temporomandibular joint and neck.

The positions of the disc and condyle were measured on radiological images both before and after treatment, and the changes in the positions of the left and right mandibular joints of the patients are listed in Table 8 and Table 9. There were 18 joints (11 healthy and 7 unhealthy) from 9 patients in the control group and 20 joints (7 healthy and 13 unhealthy) from 10 patients in the study group (all unhealthy joints had been diagnosed with anterior disc displacement with reduction). Regardless of the group and treatment, unhealthy joints had higher disc and condyle positions than healthy joints. After treatment, insignificant increases or decreases in the condyle and disc positions were observed in the control group. In the study group, the disc position of the joints with ADD in the *X*-axis moved backward compared to the pre-treatment MRI analysis (*p* = 0.017). The statistical analysis of joints that were detected as having a normal disc and condyle position revealed no statistically significant difference after 6 weeks of treatment in terms of the degree of disc position change on the *X*-axis and condyle position change on the X–Y axis (*p* > 0.05). However, in the study group, thickening of the posterior band of the articular disc was detected in the normal joints, which may result from the downward position change of the disc on the *Y*-axis (*p* = 0.028). There was no statistically significant difference between the groups regarding post-treatment disc position changes on the *Y*-axis and condyle position changes on the X–*Y*-axis (*p* > 0.05) (Table 9). In addition, effect size analysis between the control and study groups revealed a large effect for the disc position difference on the *X*-axis in the study group (Cohen’s d = 1.09) and a small effect for the disc position difference on the *Y*-axis and vice versa (Cohen’s d = 0.49) (Table 10). Consequently, the application of SS + OMT may lead to beneficial changes in disc positions of unhealthy joints.

## 4. Discussion

Osteopathic manipulative treatment is a type of manual medicine that assures the unity of the human body, mind, and spirit, as well as the myofascial interaction of the body parts and the self-healing mechanisms of the body. This treatment offers many benefits in curing musculoskeletal disorders by reducing low back pain, neck pain, and the symptoms of fibromyalgia. Additionally, its effects on recurrent acute otitis media, cerebral palsy, learning disorders, neurologic deficits, asthma, pneumonia, bronchiolitis, gastrointestinal disorders, and headaches have been reported [8,9]. However, in the treatment of TMDs, recent studies were far from utilizing an objective assessment or considering a randomized control group to address the underlying mechanism of the treatment, making osteopathic manipulative treatment questionable for routine application.

The current study explored the possible benefits of SS + OMT, considering clinical evaluations and quantitative radiological evaluations of the temporomandibular joint with a randomized control group. The results showed that applying SS + OMT may increase sleep quality, physical function, and general health while reducing pain related to TMD. Moreover, this application may improve cervical and mandibular motion and facilitate a cure for TMD.

The temporomandibular joint is attached to the cervical and thoracic regions of the body via neurological and biomechanical connections [14]. Any dysfunction around the joint may affect the structures in those regions, and any dysfunction in the cervical or thoracic regions, as well as the structures connected with fascial, muscular, ligamentous, and neural tissues in those regions, may lead to dysfunction within the masticatory system. In the current study, before treatment, the right and left rotations of the cervical spine were restricted, and the right and left lateral flexions of the cervical spine were constrained when compared to the normal ranges defined by the American Medical Association. After treatment, increases in rotation and lateral flexion were observed, especially in patients who received SS + OMT. This may be because osteopathic manipulative treatment in the current study consisted of interventions to treat the craniocervical system and its structural connections.

Many TMDs are accompanied by movement dysfunction of the mandible, inducing limited rotational and translational movements predominantly in the mediolateral and/or anterior–posterior axes. Consequently, reduced maximum mouth opening, lateral excursions, and protrusion are well-acknowledged signs of this disorder [28,29,30,31,32]. A threshold value of 30 mm for maximum mouth opening was reported for patients experiencing sudden or chronic closed locked symptoms due to TMDs, in whom conservative treatments, such as manual therapy, physiotherapy, and home exercise, are preferred [33,34]. In the study of Kropmans et al., a minimum increase of 5 mm in the opening after treatment was reported to be clinically important, especially for maximum mouth openings <35 mm before treatment [35]. However, an increase >3 mm is recognized as a remarkable improvement for maximum mouth opening ranging from 40 mm to 50 mm [35]. In the current study, the maximum mouth opening was initially 44.7 mm. The treatments led to a 3 mm increase on average, showing a remarkable improvement according to the definitions of Kropmans T. et. al. In addition, the application of SS + OMT led to a significant increase in the maximum mouth opening range. In contrast, in the current study, the lateral excursions and protrusion were within normal ranges before treatment, and no significant changes were observed after treatment. However, splint treatment complemented with osteopathic manipulative treatment facilitated lateral excursions, so that patients put less effort into performing these movements. Advances in maximum mouth opening and lateral excursions were possible because during osteopathic manipulative treatments, mandibular movements in all axes were evaluated with utmost care, and Grade I or II mobilizations were applied to the identified restricted movements of the mandible. In addition, intra- and extra-oral muscle relaxation techniques were applied to the masseter, lateral pterygoid, medial pterygoid, infrahyoid, and temporal muscles to reduce muscular tension, whereas post-isometric relaxation was applied around the masticatory muscles during mouth opening, mouth closing, protrusion, retrusion, and lateral excursions.

Temporomandibular joint pain affects general health status. The present pain levels of the patients were measured according to the visual analog scale (0–10 points). In the study of Kropmans et al., a general norm was defined for the pain status according to the VAS changes after treatment. In that study improvement in the pain status was deemed significant when the pain was reduced by at least 1.90 points [36]. The results of the current study show that SST reduces pain by 2.11 points on average, but SS + OMT leads to more significant improvements by reducing the pain by 2.50 points on average. Temporomandibular joint pain can be accompanied by various types of pain that adversely affect human health. The minimum clinically important differences in pain scores before and after treatment are reported to range from 1.50 to 3.20 points [37,38]. A significant decrease in pain was acknowledged for the cervical, thoracic, and lumbar regions, as well as for headache, by later treatment. The use of occlusal splints may trigger the inhibition of harmful and sensory stimuli around the cervical tissues and decrease cervical pain in patients with cervical dystonia [28]. However, further reductions in pain scores are achievable when additional relaxation therapies are delivered [39]. The results of the current study are in accordance with these studies, indicating that SST leads to decreased cervical pain. Meanwhile, the current study also revealed that SS + OMT may decrease cervical pain even more, while also significantly decreasing other types of pain. On the contrary, TMDs can be accompanied by sleep problems, while low sleep quality can be linked to poor treatment outcomes or non-response to treatment. Quality can be assessed using subjective sleep quality, sleep latency, habitual sleep efficiency, and sleep disturbances [40,41]. In the current study, an average of 0.11 points was noted for patients receiving SST, whereas an average of 2.30 points was observed for patients receiving SS + OMT. SS + OMT may improve sleep quality better than SST.

Self-reported measures of health from a healthy population within the 18–44-year age group point to average scores of 94.7 for physical functioning, 96.5 for social functioning, 95.3 for role limitation due to physical problems, 95.8 for role limitation due to emotional problems, 95.8 for mental health, 68.6 for energy and vitality, 90.1 for bodily pain, and 77.3 for the general perception of health [42]. In addition, a five-point change in the 0–100 scale (5%) with a moderate effect size (0.50–0.79) was determined as a minimal clinically important change in SF-36 sub-domains among patients with orthopedic problems. In the current study, lower scores were obtained for patients within the same age group, as they all had TMDs. However, the largest distinction was in the role limitation due to emotional problems score, which was 50.0, and the smallest distinction was in the energy and vitality score, which was 13.9. SST and SS + OMT increase the role limitation due to the emotional problems score, but the latter leads to a higher increase that can be clinically meaningful.

MR images of the temporomandibular joints have been used to assess disc and condyle positions to quantify the outcomes of different splint treatment plans for curing TMDs. The joints diagnosed with anterior disc displacement with reduction may gain normal disc–condyle positions when the splint is worn. Still, most discs and condyles return to their initial positions after the occlusal splint is removed [28]. On the contrary, both anterior repositioning splint treatment and stabilization splint treatment may enhance the forward and downward movements of the condyle. In contrast, the anterior repositioning splint treatment achieves better backward movements for the discs of the joints with anterior disc displacement with reduction [43]. Immediate use of an occlusal splint may also lead to 0.75 mm replacements for the disc in the backward direction [44]. To the best of our knowledge, the current study pioneered using MR images to quantify the changes in disc and condyle positions due to SS + OMT. The results showed that the treatment plan revealed better backward movements for the discs of the joints with anterior disc displacement with reduction when osteopathic treatment was added.

The current study has some limitations. The study dataset consisted of a small number of patients and joints, and more benefits could be identified when more patients and joints are considered. However, the strong Cohen’s d index finding (d = 1.09) for disc position changes in the X–Y axis suggests that osteopathic manipulative treatment is slightly effective in changing the disc position posteriorly. To understand the effectiveness of osteopathic manipulative treatment for condyle position differences, there should be more participants. Tailored splints customized to having full functionality are dedicated to use during sleep only, and 24 h use of the splints may further increase the benefits on condyle position by changing the teeth contacts in between the upper and lower jaw. An osteopathic manipulative treatment plan focusing on individual demands and considering many regions of the body in addition to the temporomandibular joint was used, and different plans targeting different body regions may lead to dissimilar outcomes. The temporomandibular joint was imaged using dedicated MR protocols, while the jaw was kept still using an MR-compatible device developed by our team. The use of different imaging protocols and jaw-fixing devices may result in different locations of the condyle and disc of the temporomandibular joint. The condyle and disc positions were measured from MR images using Drace–Enazmann’s methodology, and different methodologies may lead to different measurements.

Further assessment methods, such as computerized TMJ screening methods, could increase the effectiveness of dental treatment. Wieckiewicz et al. noted the significance of virtual assessment methods such as Gerber Dynamic Facebow in their study. Based on their results, they suggested the risks of hand measuring. Our study used the hand-measuring method for the splint appliance, which may have decreased the therapeutic effect in all groups [45].

## 5. Conclusions

In treating TMDs, interdisciplinary treatments involving dentists, osteopaths, and physiotherapists are being increasingly accepted. Preferring SS + OMT to SST may increase sleep quality, physical function, and general health while reducing pain related to TMD. Moreover, the application of osteopathic manipulative treatments may also lead to improvements in cervical and mandibular motions, which are beneficial in curing TMDs. In our study, we found significant differences in the health quality of the patients in the study group. However, patients may have been affected by their health conditions, and osteopathic treatment also involves hormonal systems. Another limitation of this study was the small sample size. Therefore, further prospective studies with a larger number of patients are needed to clarify the potential benefits of this treatment in clinical practice.

## Figures and Tables

**Table 1:** Steps involved in osteopathic treatment.

Step	Details
1	Treatment of all underlying muscular, fascial, osseous and visceral dysfunctions in the body that impair the function of the jaw joint and the postural patterns.
2	Treatment of the occipital bone and temporal bone.
3	Treatment of the masticatory muscles.
4	Treatment of the hyoid muscles.
5	Treatment of the neck muscles.
6	Treatment of the craniocervical fascias.
7	Treatment of the condyles and disks. A reduction in the intensity of pain in the facilitated segment should normally be achieved before the alignment of the TMJ is normalized.
8	Treatment of the mandibular ligaments.
9	Treatment of sphenopetrosal ligament at the sphenopetrosalsynchondrosis.
10	Improvement of nasal breathing: treatment of the paranasal sinuses and tonsils.

**Table 2:** Demographic data.

		Control Group	Study Group	*p*
Age (years)		28.00 ± 7.40	26.00 ± 6.60	0.484 ^a^
Height (m)		1.70 ± 0.08	1.70 ± 0.06	0.773 ^a^
Weight (kg)		68.00 ± 16.11	68.30 ± 13.06	0.756 ^a^
Gender	Female Male	7 (77.8%) 2 (22.2%)	9 (90.0%) 1 (10.0%)	0.582 ^b^
Smoking	No Yes	5 (55.6%) 4 (44.4%)	7 (70.0%) 3 (30.0%)	0.650 ^b^
Smoking (cigarettes/day)		3.78 ± 5.50	6.00 ± 10.75	0.850 ^a^
Smoking period (years)		3.00 ± 4.92	4.00 ± 6.82	0.850 ^a^
Alcohol Consumption	Never Occasionally Regularly (<10 years) Regularly (≥10 years)	3 (33.3%) 6 (66.7%) - -	3 (30.0%) 4 (40.0%) 2 (20.0%) 1 (10.0%)	0.527 ^c^
Disease history	No Yes	8 (88.9%) 1 (11.1%)	3 (30.0%) 7 (70.0%)	0.020 ^b^
Regular medication	No Yes	9 (100.0%) -	7 (70.0%) 3 (30.0%)	0.211 ^b^
Exercise habit	No <3 times/week ≥3 times/week	3 (33.3%) 6 (66.7%) -	4 (40.0%) 4 (40.0%) 2 (20.0%)	0.459 ^c^

^a^ Mann–Whitney U test, ^b^ Fisher exact test, and ^c^ Fisher–Freeman–Halton test.

**Table 3:** Clinical findings.

		Control Group	Study Group	*p*
Click Opening	Right joint	-	1 (10%)	0.402
	Left joint	4 (44%)	1 (10%)	
	Both	4 (44%)	6 (60%)	
	None	1 (11%)	2 (20%)	
Occlusion	Class 1	5 (55%)	9 (90%)	0.076
	Class 2	1 (11%)	-	
	Class 1 left and Class 2 right	-	1 (10%)	
	Class 2 left and Class 1 right	3 (33%)	-	
Bruxism	Present	5 (55%)	7 (70%)	0.430
	Absent	4 (45%)	3 (30%)	

**Table 4:** Pain scores.

		Control Group	Study Group	*p* ^a^	*d*
TMJ Pain	Pre Post *p* ^b^ change	4.22 ± 1.48 2.11 ± 1.69 0.011 −2.11 ± 1.62	3.30 ± 2.11 0.80 ± 1.14 0.007 −2.50 ±1.72	0.381 0.048 0.589	0.23
Cervical Pain	Pre Post *p* ^b^ Change	4.00 ± 3.04 3.11 ± 3.02 0.216 −0.89 ±1.96	3.70 ± 3.13 2.10 ± 2.13 0.033 −1.60 ± 2.27	0.837 0.382 0.472	0.33
Thoracic Pain	Pre Post *p* ^b^ Change	3.11 ± 3.22 3.67 ± 3.20 0.496 0.56 ± 2.01	3.90 ± 3.07 1.50 ± 1.43 0.024 −2.40 ± 2.59	0.584 0.183 0.018	1.27
Back Pain Pre	Post *p* ^b^ Change	4.44 ± 2.79 4.00 ± 2.96 0.317 −0.44 ± 1.24	3.40 ± 3.27 2.00 ± 2.40 0.027 −1.40 ± 1.65	0.429 0.120 0.190	0.65
Pelvic Pain	Pre Post *p* ^b^ Change	1.00 ± 2.12 0.78 ± 1.72 0.593 −0.22 ± 1.30	1.20 ± 1.75 0.50 ± 1.27 0.066 −0.70 ± 1.06	0.586 0.819 0.320	0.41
Headache	Pre Post *p* ^b^ Change	4.00 ± 2.74 3.11 ± 2.03 0.194 −0.89 ± 2.03	3.20 ± 3.36 0.70 ± 1.64 0.027 −2.5 ± 2.88	0.646 0.016 0.180	0.64

^a^ Mann–Whitney U test ^b^ Wilcoxon sign test. *d* = effect size.

**Table 5:** Quality of life data.

		Control Group	Study Group	*p* ^a^	*d*
Physical Function	Pre Post *p* ^b^ Change	81.67 ± 15.21 83.89 ± 15.96 0.796 2.22 ± 11.21	80.50 ± 18.48 92.00 ± 10.85 0.018 11.5 ± 11.07	0.967 0.182 0.048	0.83
Social Function	Pre Post *p* ^b^ Change	70.83 ± 20.73 66.67 ± 27.95 0.496 −4.17 ± 18.75	72.50 ± 18.45 83.75 ± 15.65 0.056 11.25 ± 16.08	0.799 0.139 0.096	0.89
Role Physical	Pre Post *p* ^b^ Change	77.78 ± 29.17 75.00 ± 30.62 0.832 −2.78 ± 44.10	65.00 ± 47.43 65.00 ± 31.62 0.891 0.00 ± 39.09	0.715 0.548 0.609	0.10
Role Emotional	Pre Post *p* ^b^ Change	37.03 ± 42.31 48.14 ± 50.31 0.461 11.11 ± 55.28	46.66 ± 42.17 60.01 ± 43.89 0.260 13.35 ± 42.16	0.579 0.727 0.932	0.50
Mental Health	Pre Post *p* ^b^ Change	63.11 ± 11.62 69.78 ± 11.68 0.203 6.67 ± 13.11	69.60 ± 6.02 76.00 ± 10.67 0.073 6.40 ± 10.36	0.134 0.174 0.622	0.20
Energy and Vitality	Pre Post *p* ^b^ Change	53.89 ± 15.16 53.89 ± 22.75 1.000 0.00 ± 12.50	56.50 ± 14.15 65.50 ± 18.92 0.073 9.00 ± 13.90	0.805 0.324 0.150	0.68
Bodily Pain	Pre Post *p* ^b^ Change	73.89 ± 18.80 80.56 ± 16.05 0.034 6.67 ± 7.07	67.25 ± 24.25 81.75 ± 15.86 0.188 14.50 ± 30.00	0.559 0.798 0.276	0.36
General Health	Pre Post *p* ^b^ Change	62.78 ± 13.72 70.56 ± 16.67 0.065 7.78 ± 11.21	61.00 ± 21.96 72.00 ± 17.83 0.027 11.00 ± 12.87	0.560 0.902 0.740	0.27

^a^ Mann–Whitney U test. ^b^ Wilcoxon sign test. *d* = effect size.

**Table 6:** Sleep quality.

		Control Group	Study Group	*p* ^a^	*d*
Sleep Quality	Pre Post *p* ^b^ Change	6.11 ± 2.62 6.00 ± 3.35 0.892 −0.11 ± 4.48	8.60 ± 2.59 6.30 ± 3.77 0.018 −2.30 ± 2.06	0.047 0.804 0.093	0.64

^a^ Mann–Whitney U test ^b^ Wilcoxon sign test. *d* = effect size.

**Table 7:** The maximum range of motions.

		Control Group	Study Group	*p* ^a^	*d*
Mouth	Pre	44.00 ± 6.56 46.67 ± 4.69 0.176 2.67 ± 5.39	44.70 ± 6.98 47.70 ± 4.90 0.027 3.00 ± 3.97	0.710 0.650 0.524	0.07
Opening (mm)	Post *p* ^b^ Change				
Mandibular	Pre	4.22 ± 1.56 4.56 ± 1.51 0.257 0.33 ± 0.87	4.80 ± 1.55 5.10 ± 0.99 0.429 0.30 ± 1.16	0.394 0.421 0.829	0.17
Protrusion (mm)	Post *p* ^b^ Change				
Mandibular	Pre	4.78 ± 2.44 4.56 ± 1.74 0.414 −0.22 ± 0.83	3.60 ± 1.43 5.30 ± 1.77 0.011 1.70 ± 1.34	0.403 0.331 0.002	1.70
Retrusion (mm)	Post *p* ^b^ Change				
Mandibular Right	Pre	8.67 ± 2.96 8.44 ± 3.40 0.732 −0.22 ± 1.72	8.20 ± 1.55 8.20 ± 2.78 0.674 0.00 ± 3.30	0.483 0.362 0.454	0.09
Lateral Excursion (mm)	Post *p* ^b^ Change				
Mandibular Left	Pre	9.00 ± 2.18 9.44 ± 2.65 0.395 0.44 ± 1.51	6.50 ± 1.43 8.30 ± 1.25 0.011 1.80 ± 1.62	0.030 0.112 0.087	0.86
Lateral Excursion (mm)	Post *p* ^b^ Change				
Cervical Right	Pre	81.00 ± 8.85 79.56 ± 6.25 0.446 1.44 ± 8.68	80.90 ± 10.04 84.40 ± 5.83 0.072 3.50 ± 6.65	0.651 0.090 0.158	0.26
Rotation (°)	Post *p* ^b^ Change				
Cervical Left	Pre	80.67 ± 7.98 77.56 ± 6.95 0.183 −3.11 ± 7.15	79.00 ± 9.70 85.40 ± 4.20 0.008 6.40 ± 7.12	0.743 0.024 0.005	1.33
Rotation (°)	Post *p* ^b^ Change				
Cervical Right	Pre	22.44 ± 5.57 22.56 ± 5.64 0.317 0.11 ± 0.33	24.20 ± 3.19 26.60 ± 3.34 0.027 2.40 ± 2.91	0.671 0.226 0.021	1.08
Lateral Flexion (°)	Post *p* ^b^ Change				
Cervical Left	Pre	23.56 ± 5.83 23.33 ± 5.87 0.581 −0.22 ± 1.48	23.90 ± 4.12 27.20 ± 3.71 0.018 3.30 ± 3.80	0.935 0.212 0.012	1.19
Lateral Flexion (°)	Post *p* ^b^ Change				

^a^ Mann–Whitney U test. ^b^ Wilcoxon sign test. Cohen’s *d* = effect size.

**Table 8:** Disc and condyle positions for the joints.

		Normal Joint		*p* ^a^	ADD Joint		*p* ^a^
		Control Group (*n* = 11)	Study Group (*n* = 7)		Control Group (*n* = 7)	Study Group (*n* = 13)	
Disc	Pre, x	0.84 ± 0.50	1.36 ± 0.36	0.023	−3.87 ± 2.07	−5.16 ± 1.97	0.362
	y	−2.47 ± 0.31	−2.21 ± 0.15	0.103	−4.87 ± 1.61	−6.11 ± 1.42	0.285
	Post, x	0.85 ± 0.50	1.39 ± 0.32	0.021	−3.92 ± 2.16	−4.42 ± 1.45	
	y	−2.49 ± 0.36	−2.41 ± 0.30	0.683	−4.96 ±1.55	−6.06 ± 1.49	
	Change, x	0.01 ± 0.09	0.03 ± 0.12	0.440	−0.05 ± 0.42	0.74 ± 0.80	0.088
	y	−0.11 ± 0.35	−0.25 ± 0.46	0.964	−0.00 ± 0.17	0.20 ± 0.57	0.721
Condyle	Pre, x	−0.20 ± 0.52	−0.03 ± 0.68	0.319	−0.38 ± 1.05	0.55 ± 0.84	0.721
	y	−7.28 ± 0.81	−6.65 ± 0.46	0.113	−6.72 ± 1.06	−5.89 ± 1.42	0.143
	Post, x	−0.32 ± 0.68	−0.27 ± 0.90	0.856	−0.38 ± 1.02	0.75 ± 0.73	
	y	−7.36 ± 0.83	−6.52 ± 0.92	0.113	−6.60 ± 0.99	−5.98 ± 1.24	
	Change, x	0.02 ± 0.13	−0.20 ± 0.29	0.041	−0.09 ± 0.32	0.06 ± 0.60	0.552
	y	−0.08 ± 0.25	0.13 ± 0.70	0.856	0.12 ± 0.29	−0.09 ± 0.57	0.552

^a^ Mann-Whitney U test.

**Table 9:** Comparison of the mean disc coordinates between groups for normal joints and those with ADDwR.

Coordinates		Normal Joints (X,Y)	ADD Joints (X,Y)
Splint Group	Before Treatment	(0.84 ± 0.50, −2.47 ± 0.31)	(−3.87 ± 2.07, −4.87 ± 1.61)
	After Treatment	(0.85 ± 0.50, −2.49 ± 0.36)	(−3.92 ± 2.16, −4.96 ± 1.55)
OMT + Splint Group	Before Treatment	(1.36 ± 0.36, −2.21 ± 0.15 ^b^)	(−5.16 ± 1.97 ^a^, −6.11 ± 1.42)
	After Treatment	(1.39 ± 0.32, −2.41 ± 0.30 ^b^)	(−4.42 ± 1.45 ^a^, −6.06 ± 1.49)

^a^ In the OMT + Splint Group, the disc position of the joints with ADD in *X*-axis moved backward when compared to before treatment, *p* = 0.017. ^b^ In the OMT + Splint Group the disc position of normal joints in *Y*-axis moved downward when compared to before treatment, *p* = 0.028.

**Table 10:** Power analysis of the effect of OMT + splint treatment differences on disc and condyle positions.

	Effect Size (d)
Disc position difference in *X*-axis (mm)	1.09
Disc position difference in *Y*-axis (mm)	0.49
Condyle position difference in *X*-axis (mm)	0.06
Condyle position difference in *Y*-axis (mm)	0.06

d = 0.2–0.5; small. d = 0.51–0.80; medium. d > 0.80 large effect. d > 1 very large effect.

## Data Availability

Data is unavailable due to privacy and ethical restrictions.
